# Spontaneous Tumor Lysis Syndrome in Diffuse Large B-cell Lymphoma: Early Diagnosis and Management

**DOI:** 10.7759/cureus.4679

**Published:** 2019-05-16

**Authors:** Mounika Gangireddy, Isha Shrimanker, Vinod K Nookala, Kathryn A Peroutka

**Affiliations:** 1 Internal Medicine, University of Pittsburgh Medical Center - UPMC - Pinnacle, Harrisburg, USA; 2 Hematology-Oncology, Andrews and Patel Associates, Harrisburg, USA

**Keywords:** spontaneous tumor lysis syndrome, allopurinol, rasburicase, hyperuricemia, renal failure

## Abstract

Spontaneous tumor lysis syndrome is a rare oncological emergency associated with multiorgan failure. It is characterized by an elevation of uric acid, hyperphosphatemia, hypocalcemia, hyperkalemia and renal failure in the setting of no active chemotherapy as a result of lysis of massive tumor burden. Early recognition of the disease and prompt management would affect morbidity and mortality. We present the case of an 80-year-old Caucasian male with a history of recently diagnosed diffuse large B-cell lymphoma who had worsening fatigue, weakness and decreased appetite for three days. On admission, laboratory investigations were significant for elevated creatinine, uric acid, and phosphorous. He was started on hemodialysis and rasburicase in view of hyperuricemia. Subsequently, chemotherapy was started. He tolerated chemotherapy initially but later developed multiorgan failure. His family then opted for comfort measures and the patient passed away soon after. In conclusion, spontaneous tumor lysis syndrome is a common association with hematological cancers. Prophylaxis with allopurinol and rasburicase is recommended in all patients who are at an increased risk for tumor lysis syndrome.

## Introduction

Tumor lysis syndrome (TLS) is considered an emergency in the field of oncology. In 1929, Bedrna and Polcák reported the first case of TLS in patients with chronic leukemia who underwent irradiation [1]. It occurs when there is a large release of intracellular components into the systemic circulation, leading to multiple electrolyte derangements as a result of the massive tumor burden. These include hyperuricemia, hyperkalemia, hyperphosphatemia, and hypocalcemia [2]. TLS usually occurs when cytotoxic chemotherapy is started in patients with hematologic malignancies [3].

Hyperkalemia can lead to life-threatening arrhythmias. Hyperphosphatemia can lead to tetany and seizure precipitation due to secondary hypocalcemia. Hyperuricemia can lead to acute kidney injury. A systemic inflammatory response syndrome may result due to the release of cytokines when tumor lysis occurs. All these manifestations can eventually lead to multiple organ failure [4].

Spontaneous tumor lysis syndrome (SPTLS) can be described when TLS is manifested in the absence of active chemotherapy [5]. There is little literature to prove the exact mechanism of SPTLS. Previous studies have suggested that a high formation of glucocorticoids and also hyperthermia may result in tumor cell death [6].

It has been reported that SPTLS occurs in malignancies including Burkitt’s lymphoma [7], acute myeloid leukemia [8], anaplastic large T cell lymphoma [9], myelofibrosis [10], and diffuse large B cell lymphoma along with Richter syndrome [11].

We report the presentation and management of spontaneous tumor lysis syndrome in a case of diffuse large B-cell lymphoma.

## Case presentation

An 80-year-old Caucasian male with a past medical history of hypertension and hyperthyroidism was evaluated for increasing fatigue and weakness with loss of appetite for three days.

**Figure 1:**
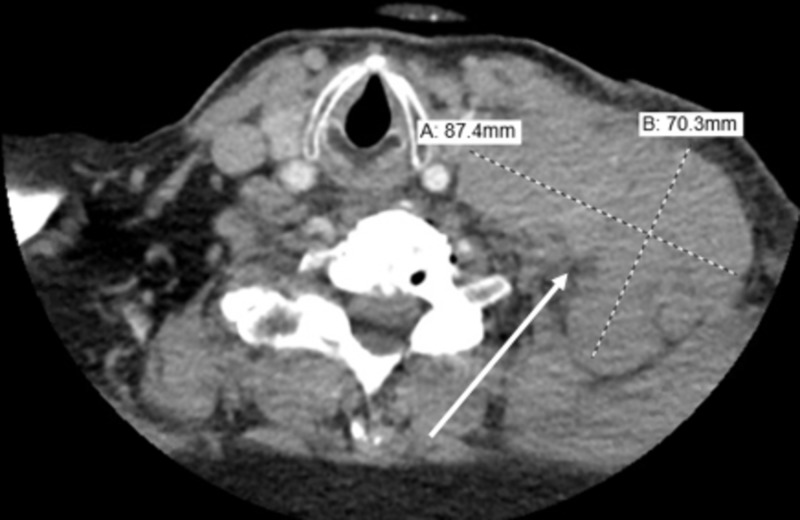
Computed tomography of the soft tissue of neck revealing an 8.7 x 6 cm soft tissue mass.

The patient had complaints of an enlarging left neck mass that started a couple of months ago. Computed tomography of the neck revealed the presence of an 8.7 x 6 cm soft tissue mass in the mid-left cervical region extending caudally in the left supraclavicular and infraclavicular regions (Figure 1). He underwent a biopsy of the mass which showed diffuse large B-cell lymphoma with t(14;18) translocation. He was undergoing a staging workup in anticipation of chemotherapy with mini-CHOP [cyclophosphamide, doxorubicin hydrochloride, vincristine sulfate (Oncovin), prednisone] plus rituximab, commonly known as R-miniCHOP. It was further complicated by deep vein thrombosis in the right internal jugular, basilic, and brachial vein, and he was started on apixaban.

**Figure 2:**
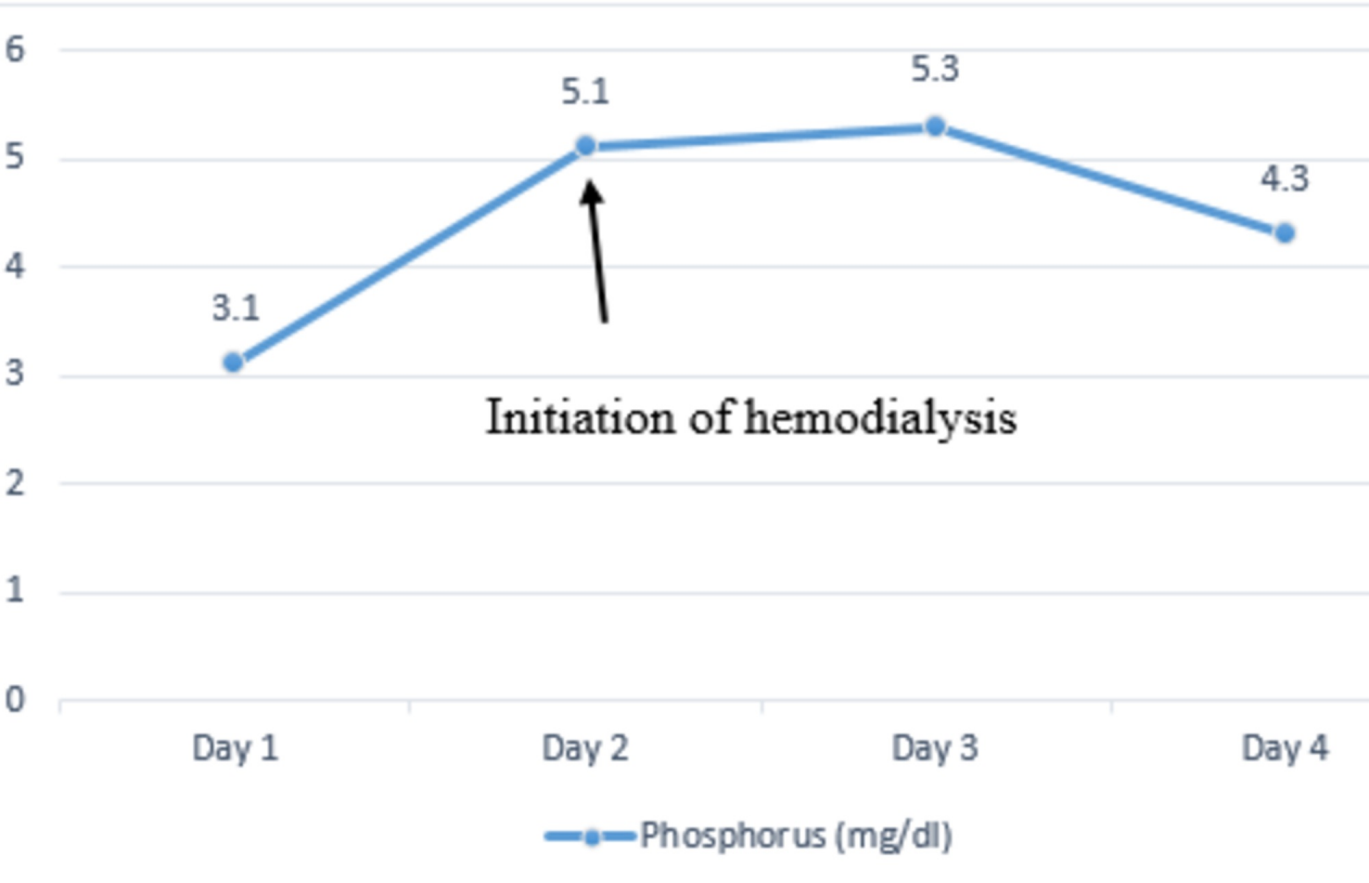
Trends of serum phosphorus during the hospital stay.

**Figure 3:**
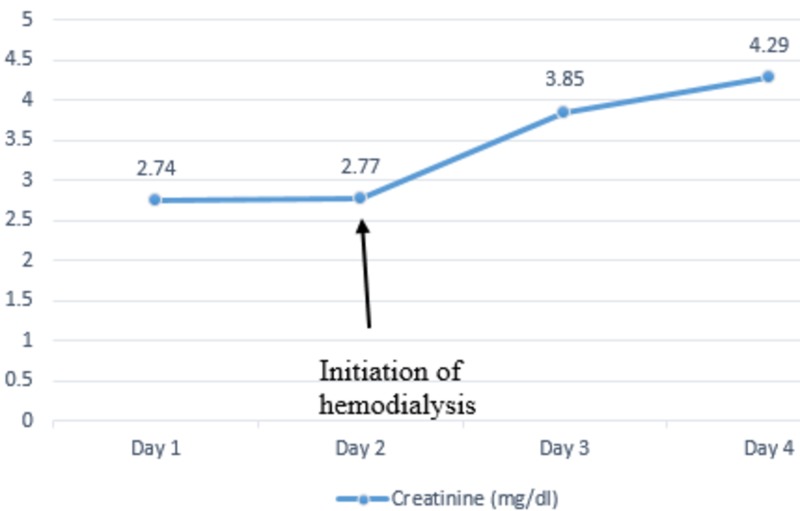
Trends of serum creatinine during the hospital stay.

**Figure 4:**
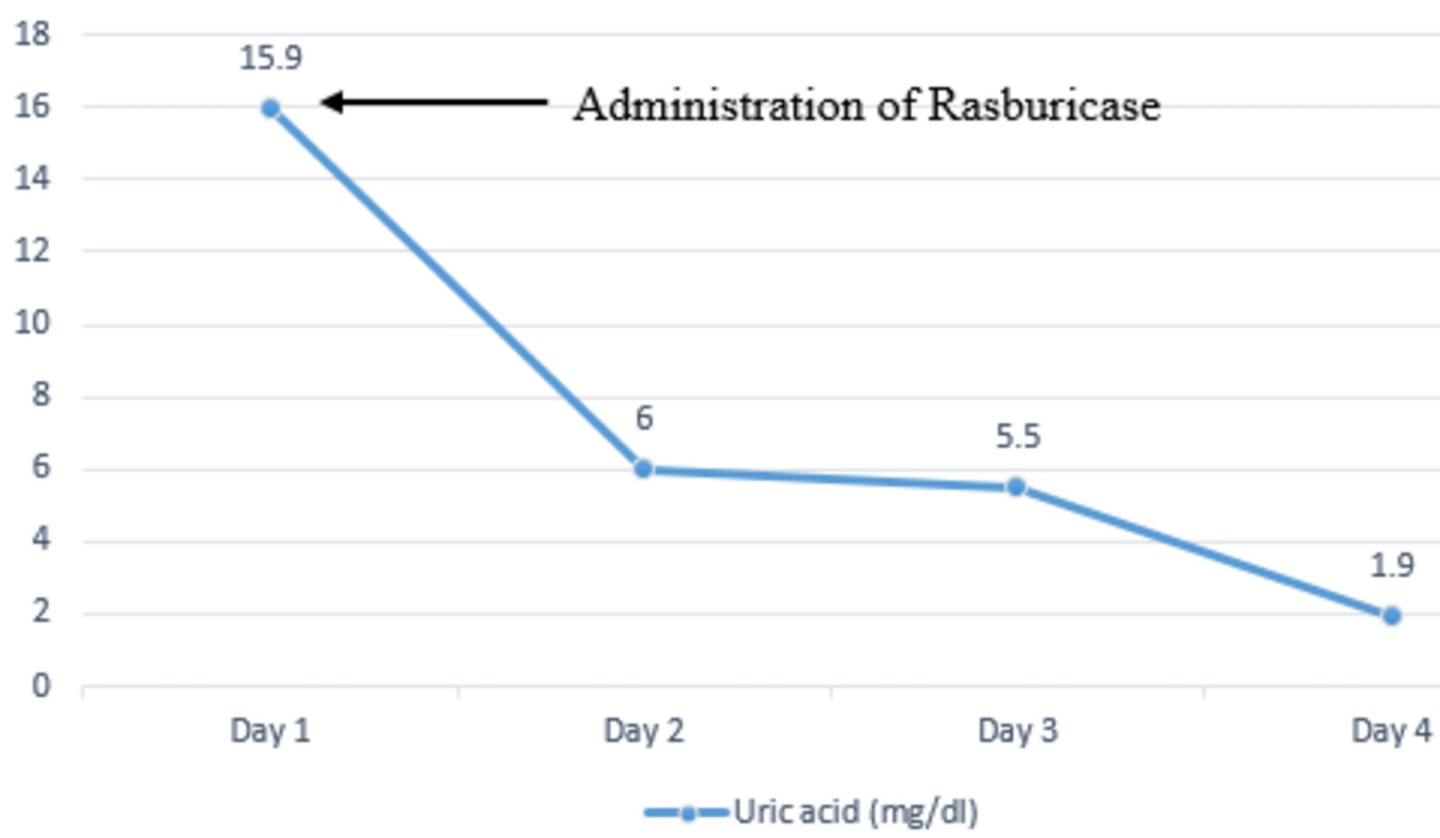
Trends of serum uric acid during the hospital stay.

On admission, his vital signs were a pulse of 83 beats per minute, respiratory rate of 24 breaths per minute, blood pressure of 122/63 mm of Hg, temperature of 36.3 °C and oxygen saturation of 100% on room air. Physical exam showed diffuse cervical lymphadenopathy with a left neck mass measuring 9 x 6 cm. Laboratory investigations were significant for elevated creatinine of 2.74 mg/dl (baseline of 0.7 mg/dl), uric acid of 15.9 mg/dl and phosphorous of 5 mg/dl with lactate dehydrogenase (LDH) of 800 U/l. His potassium levels were 3.7 mmol/l, well within the normal range. He was started on aggressive hydration and was given a dose of rasburicase with improvement in uric acid levels from 15.9 mg/dl to 6.0 mg/dl the following day. Despite aggressive resuscitation, he had a decrease in urine output and subsequently developed acute renal failure requiring hemodialysis. The trends of serum phosphorus (Figure 2), serum creatinine (Figure 3), and serum uric acid (Figure 4) are shown respectively.

He was then started on chemotherapy with R-miniCHOP regimen. He was started on allopurinol for prophylaxis of tumor lysis syndrome in the setting of massive tumor burden. He developed severe pancytopenia as a result of chemotherapy and eventually developed multi-organ failure in the setting of poor prognosis. His family opted for comfort measures and the patient passed away soon after.

## Discussion

In any type of cancer, SPTLS is a rare occurrence. A study that was conducted by Tsokos et al. revealed that out of the 33 patients that were diagnosed with non‐Hodgkin lymphoma, three of them had increased levels of uric acid and renal failure before the start of therapy. Their blood work revealed plasma uric acid concentration >17 mg/dL (1012 μmol/L) and acute renal failure that required immediate hemodialysis, even before the start of chemotherapy [12].

The risk factors for SPTLS can be divided into the intrinsic nature of the tumor and extrinsic factors associated with the host. Tumor-related intrinsic factors include an increased proliferation rate [13] with a rise in the Ki-67 score, sensitivity to chemotherapy and radiation, size of the tumor >10 cm in diameter, white blood cell count >50 000/μL, LDH levels that are twice the normal range and infiltration of the bone marrow. Our patient had a highly proliferative tumor, LDH levels that were two times the upper normal limit and anticipated to undergo chemotherapy with R-miniCHOP, resulting in a predisposition to SPTLS. At the same time, host-related extrinsic factors such as uric acid of 15.9 mg/dL, a significant elevation in serum creatinine of 2.74 mg/dL (when his baseline was 0.7 mg/dL), further contribute to the development of SPTLS. Cairo and Bishop had formulated a combination of laboratory and clinical criteria for the diagnosis of TLS, for which two of the laboratory findings should be present for three days before the initiation of therapy or seven days after therapy and at least one clinical criteria should be present (Tables 1, 2) [13].

**Table 1:** Laboratory criteria.

Laboratory investigation	Value	Change from baseline value
Uric acid	≥ 476 μmol/mL (> 8 mg/dL)	Increase by 25%
Phosphorus	≥ 1.45 mmol/L (> 4.5 mg/dL)	Increase by 25%
Potassium	≥ 6.0 mmol/L (> 6 mEq/L)	Increase by 25%
Corrected calcium	≤ 1.75 mmol/L (<7 mg/dl)	Decrease by 25%

**Table 2:** Clinical criteria.

Laboratory investigation	Grade 0	Grade 1	Grade 2	Grade 3	Grade 4	Grade 5
Creatinine	None	1.5 times upper limit of normal (ULN)	> 1.5-3.0 times ULN	> 3.0-6.0 times ULN	> 6.0 times ULN	Death
Cardiac arrhythmia	None	Intervention not indicated	Non-urgent medical intervention indicated	Symptomatic and incompletely controlled medically or controlled with a device (e.g., defibrillator)	Life-threatening complications (e.g., shock, arrhythmia in association with heart failure, hypotension, syncope)	Death
Seizure	None	-	One brief, generalized seizure; seizure(s) well controlled by anticonvulsants or infrequent focal motor seizures not interfering with activities of daily living	Seizure in which there is an altered level of consciousness; poorly controlled seizure disorder; with breakthrough generalized seizures despite medical treatment	Seizure of any kind which is prolonged, repetitive or difficult to control (e.g., status epilepticus, intractable epilepsy)	Death

It has been proposed that SPTLS can present with hyperuricemia in the absence of hyperphosphatemia. The rapid growth of the tumor due to increased cellular proliferation results in a rise in the serum uric acid levels due to a rapid proliferation of nuclear proteins. However, the phosphorus that is released is reutilized for the formation of new tumor cells [14]. On the contrary, TLS that occurs after the initiation of therapy results in rapid cell destruction with phosphorus reuptake and leads to hyperphosphatemia.

In SPTLS, at times no electrolyte abnormality may be seen until overt TLS develops. Two separate theories have been suggested for such an acute occurrence, cells undergoing necrosis result in the release of intracellular contents that are then reabsorbed into the proliferating cells [15] and that the electrolyte abnormality is compensated by renal clearance [16].

**Figure 5:**
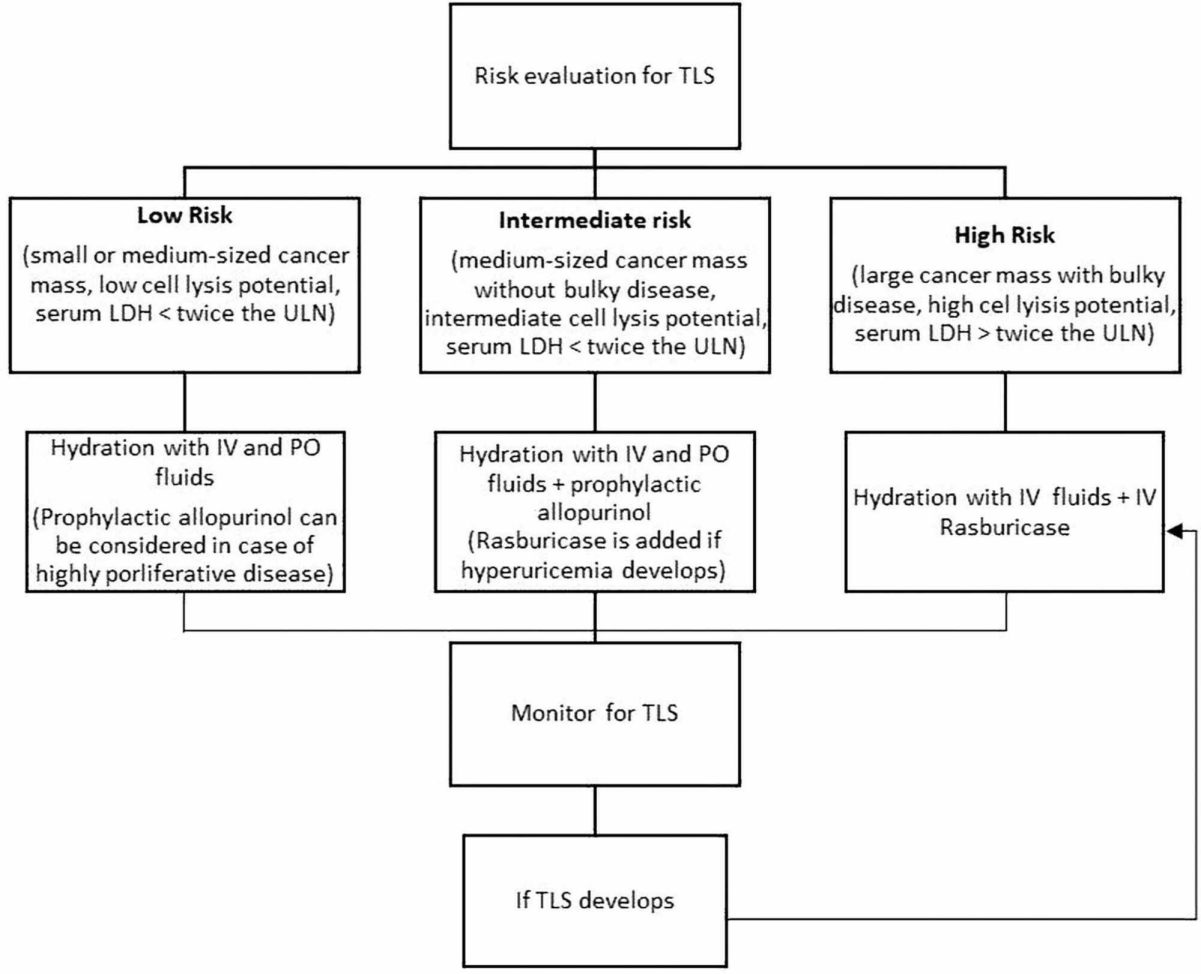
Risk evaluation and management of TLS. TLS: Tumor Lysis Syndrome; ULN: Upper Limit of Normal; LDH: Lactate Dehydrogenase; IV: Intravenous; PO: Per Oral.

Prophylactic therapy is crucial for TLS management (Figure 5). Coiffier et al. reported that aggressive intravenous fluid therapy, which is almost twice the daily fluid maintenance, should be the initial management unless contraindications for fluid therapy are present. This helps in the removal of uric acid and phosphate from circulation [3]. In patients who are at high risk for development of TLS, prophylactic rasburicase is preferred as compared to allopurinol, since the latter primarily stops the formation of new uric acid but does not affect the existing levels in blood [16]. As for the patients that are at intermediate risk of development of TLS, allopurinol is preferred as compared to rasburicase if there is no hyperuricemia [3]. Moreover, allopurinol should be used with great caution in patients with abnormal kidney function, since it is excreted via the kidney. Hemodialysis is the preferred therapy in patients who have developed TLS, as it effectively normalizes the electrolyte derangement and results in rapid excretion of uric acid [13].

Studies have revealed an increased efficacy of rasburicase when compared to allopurinol for the treatment of hyperuricemia in patients with hematologic cancers. Coiffier et al. conducted a study which revealed that initiation of rasburicase to control hyperuricemia helped in the prevention of TLS, in patients who were diagnosed with aggressive non-Hodgkin's lymphomas [17].

## Conclusions

Thus, in conclusion, SPTLS occurs rarely but has fatal consequences. Clinicians should be wary about the intrinsic and extrinsic risk factors with special attention to malignancies with a high proliferation rate. Our case reiterates the need for early recognition of this syndrome and initiation of appropriate therapy.
